# Associations of the Need for Surgery in Emergency Department Patients with Small Bowel Obstructions

**DOI:** 10.5811/westjem.18455

**Published:** 2024-11-19

**Authors:** Daniel J. Berman, Alexander W. Mahler, Ryan C. Burke, Andrew E. Bennett, Nathan I. Shapiro, Leslie A. Bilello

**Affiliations:** * Beth Israel Deaconess Medical Center, Department of Emergency Medicine, Boston, Massachusetts; † University of Southern Denmark, Odense, Denmark; ∘ Co-first authors

## Abstract

**Objectives:**

Management strategies for small bowel obstruction (SBO) vary from conservative approaches to surgical intervention. A known complication of surgery is the subsequent adhesions that can cause recurrent SBOs, longer hospital stays, and higher treatment costs. Our primary outcome was to identify independent risk factors that are associated with the decision for surgical intervention, and our secondary outcome was to describe characteristics of visits associated with complications.

**Methods:**

This study was a single-center, retrospective chart review from a large, urban university hospital. We included adult patients admitted to the emergency department (ED) with the International Classification of Diseases, 10^th^ Rev, codes for small bowel obstruction from June 1, 2017– May 30, 2019. Eligible covariates were demographics, radiological findings, clinical presentation, past medical history, and results of radiologic testing. We identified univariate associations of outcome and then performed a multivariate logistic regression to identify independent associations of each outcome. Finally, a backwards selection was used to determine the final model. We calculated odds ratios (OR) and 95% confidence intervals (CI) along with the area under the curve (AUC), as appropriate.

**Results:**

A total of 530 patients met the study criteria; 148 (27.9%) underwent surgery of whom 35 (6.6%) had complications. We identified seven independent associations for the decision of surgery: abdominal distension (OR 0.27, 95% CI 0.10–0.62); gastrografin (OR 0.41, 95% CI 0.20–0.81); previous SBO (OR 0.42, 95% CI 0.26–0.66); higher Charlson Comorbidity Index score (OR 0.87, 95% CI 0.80–0.95); nasogastric decompression (OR 2.04, 95% CI 1.25–3.39), initial systolic blood pressure <100 mm Hg (OR 2.65, 95% CI 1.05–6.53); free fluid or volvulus/closed-loop obstruction on computed tomography (OR 7.95, 95% CI 4.25–15.39), with the AUC for the predictive model equaling 0.73.

**Conclusion:**

We identified seven independent associations present in the ED associated with the decision for surgery. These associations are a step toward building better prediction models and improving decision-making in the ED, allowing for a more adequate treatment plan.

Population Health Research Capsule What do we already know about this issue?

*There are varying management strategies for small bowel obstructions (SBO), but it is not clear which patients would most benefit from surgical intervention.*

What was the research question?

*Are there independent risk factors associated with surgical intervention for patients with SBO? And which patients developed complications during their inpatient admission?*

What was the major finding of the study?

*Seven factors were associated with decision for surgery: abdominal distension (OR 0.27, 95% CI 0.10–0.62); gastrografin use (OR 0.41, 0.20–0.81); previous SBO (0.42, 0.26–0.66); higher Charlson Comorbidity Index (0.87, 0.80–0.95); nasogastric tube (2.04, 1.25–3.39); systolic blood pressure <100 mm Hg (2.65, 1.05–6.53); free fluid or volvulus/closed-loop obstruction on CT (7.95, 4.25–15.39).*

How does this improve population health?

*These findings can help clinicians identify patients who might be a better candidate for surgical intervention vs conservative therapy to safely manage SBOs.*

## INTRODUCTION

There is currently a shift from the traditional dogma, “*Never let the sun rise or set on small bowel obstruction,*” which implies urgent surgical intervention, toward non-operative management when clinically indicated.^1^ This is an important shift because non-operative management may have less associated risk and help decrease resource utilization when appropriate.^2^ ^–^ ^4^ The decision regarding small bowel obstruction (SBO) treatment and intervention is not always clear. While some studies have tried to elucidate ways to help manage our clinical decisions, there are no uniformly accepted clinical decision rules, and the approach is largely left to individual clinical judgment.

When evaluating a patient with a suspected SBO, it is important to understand the degree of obstruction and associated complications such as bowel ischemia, perforation, peritonitis, hernia strangulation, and anticipated course.^5^ ^,^ ^6^ These considerations are important as surgery itself carries risks such as infection, bleeding, and complications from general anesthesia. Also concerning is the risk of additional adhesions leading to recurrent SBOs, as post-surgical adhesions are responsible for roughly 70% of all SBOs in the United States.^2^ ^,^ ^3^ ^,^ ^7^ ^,^ ^8^ Conversely, it is imperative to discuss the risk of delayed intervention in SBO patients who require surgery. Examples include complications such as bowel resection, prolonged postoperative length of stay, and death.^9^ The management for each SBO patient should be tailored to the degree of obstruction, associated characteristics, and anticipated course while evaluating the risks and benefits of conservative management versus surgical intervention.

Our goal in this study was to identify clinical associations that identify patients likely to progress to a surgical intervention. We collected data on patient demographics, physical exam findings, vital sign abnormalities, laboratory test results, and radiographic findings associated with SBOs diagnosed in the ED. Our primary outcome was to identify independent risk factors associated with the decision for surgical intervention, and our secondary outcome was to identify independent factors associated with complications.

## METHODS

### Study Design

We performed a retrospective chart review of patients presenting to the ED (annual ED volume is 55,000 adult patients) of an urban, tertiary-care, academic medical center from June 1, 2017–May 30, 2019. Our study was reviewed and approved by the institutional review board. We followed all the best practices for chart review as described in Worst and Bledsoe with the exception of one, as our abstractors were not blind to the hypothesis.^10^

### Inclusion and Exclusion

The study population consisted of ED patients ≥18 years with an International Classification of Diseases, 10^th^ Rev. (ICD-10) code consistent with SBO (ICD-10 K56x, K91.3x, Q41.9, Q42.9, Q42.8, and Q43.1). We excluded patients who had advanced directives to avoid surgical intervention (ie, if the patient was a surgical candidate but elected not to have surgery) and patients with a large bowel obstruction identified by attending radiologist interpretation on computed tomography (CT).

### Data Collection and Handling

We abstracted data via chart review by trained reviewers compiling data from the initial presentation without knowledge of the subsequent hospital course. The parameters investigated included demographics, elements of the clinical presentation, past medical history, vital signs, physical exam findings, and radiographic imaging. Specific demographics and patient data included age, gender, date of admission, and length of stay. Pertinent past medical history included previous SBOs, inflammatory bowel disorders, past abdominal surgeries, anatomical anomalies, and malignancies. We also included parameters that comprise the Charlson Comorbidity Index (CCI), while excluding connective tissue diseases. We had one attending emergency radiologist review all CT images to identify the following features: presence of a transition point; free intraperitoneal fluid; debris and gas bubbles within the dilated small bowel lumen (small bowel feces sign); mesenteric edema; and closed-loop obstruction or volvulus to ensure consistent wording throughout the radiographic reports. We additionally noted whether oral, water-soluble radiological contrast, specifically gastrografin, had been used during their hospitalizations. We coded for non-operative surgical management, as well as complications such as sepsis, intubation, vasopressor-dependent shock, anatomic surgical alterations, and bowel perforation.

These data points were abstracted from ED records, inpatient hospital records, and discharge summaries. A trained, experienced researcher underwent rigorous training on our explicit protocol, including clearly defined variables and standardized coding methods, and performed the systematic data abstraction. The abstractor flagged ambiguous charts for additional review by an emergency medicine senior resident physician and a board-certified emergency attending physician.

### Outcomes of Interest

The primary outcome was surgical intervention, defined as surgery during initial hospital admission. The secondary outcome was SBO-related complications during initial hospital admission. Complications were also looked for after 90 days after discharge but using solely our hospital system health records. Complications included sepsis, intubation, vasopressor-dependent shock, anatomic surgical alterations, and bowel perforation.

### Data Analysis

We employed chi-square tests for categorial variables and *t*-tests for continuous variables to identify univariate associations of outcomes. The Fisher exact test was used instead of chi-square test when the sample number was low. Factors associated with surgical intervention were determined with a multivariate logistic regression model. We employed a multistage process to determine the covariates to include in the model. Possible associations were included in the model if the *P*-value for the univariate analysis was <0.10 and the prevalence was sufficiently high to allow for model convergence. Finally, backwards selection was used to determine the final model, with an alpha for exit of 0.05. The odds ratios (OR), 95% confidence intervals (CI), and area under the curve (AUC) were calculated.

## RESULTS

**Figure 1.**
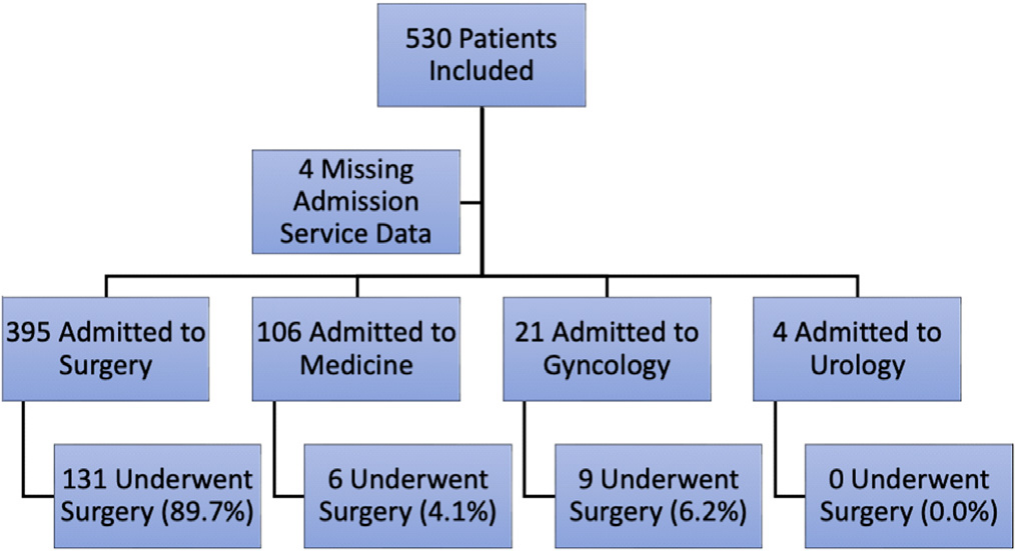
Admitting service and rates of surgery.

There were 690 patients identified and reviewed for eligibility. A total of 530 patients (76.8%) met the inclusion criteria and were included in the study. The patient population tended to be older, had a history of abdominal surgeries, and previously diagnosed SBOs (Table 1). Of the 530 eligible patients, 148 (27.9%) underwent surgery under various admitting services (Figure 1) and 35 (6.6%) had at least one complication (Table 2).

**Table 1.** Baseline characteristics of the patients included in the study, overall and by outcomes.

Characteristic	Overall (N = 530)	Surgery			Complications		
		Yes (n = 148)	No (n = 382)	*P*-value	Yes (n = 35)	No (n = 495)	*P*-value
*Demographics*							
Age, mean (SD)	63.9 (15.9)	65.1 (15.2)	63.4 (16.2)	0.28	66.5 (14.8)	63.7 (16.0)	0.32
Female, n (%)	297 (56%)	89 (60.1%)	208 (54.5%)	0.24	18 (51.4%)	279 (56.4%)	0.57
*Symptoms reported*							
Constipation, n (%)	34 (6.4%)	11 (7.4%)	23 (6.0%)	0.55	0 (0.0%)	34 (6.9%)	0.15*
Abdominal pain, n (%)	496 (93.6%)	136 (91.9%)	360 (94.2%)	0.32	32 (91.4%)	464 (93.7%)	0.48*
Abdominal distension, n (%)	68 (12.8%)	9 (6.1%)	59 (15.5%)	0.004	3 (8.6%)	65 (13.1%)	0.60*
Nausea, n (%)	325 (61.3%)	88 (59.5%)	237 (62.0%)	0.58	23 (65.7%)	302 (61.0%)	0.58
Fever, n (%)	16 (3.0%)	4 (2.7%)	12 (3.1%)	1.0000*	3 (8.6%)	13 (2.6%)	0.08*
Vomiting, n (%)	357 (67.4%)	87 (58.8%)	270 (70.7%)	0.01	25 (71.4%)	332 (67.1%)	0.60
*Medical history*							
CCI score, mean (SD)	4.2 (3.1)	3.7 (2.8)	4.5 (3.2)	0.01	5.3 (3.4)	4.2 (3.0)	0.04
Previous SBO, n (%)	263 (49.6%)	52 (35.1%)	211 (55.2%)	<0.0001	10 (28.6%)	253 (51.1%)	0.01
Crohn’s disease, n (%)	53 (10.0%)	7 (4.8%)	46 (12.0%)	0.01	2 (5.9%)	51 (10.3%)	0.56*
Ulcerative colitis, n (%)	42 (7.9%)	9 (6.1%)	33 (8.6%)	0.34	0 (0.0%)	42 (8.5%)	0.10*
Intestinal cancer, n (%)	67 (12.6%)	9 (6.1%)	58 (15.2%)	0.005	3 (8.6%)	64 (12.9%)	0.60*
Solid tumor malignancy, n (%)	195 (36.8%)	37 (25.0%)	158 (41.4%)	0.0005	15 (42.9%)	180 (36.4%)	0.44
Previous abdominopelvic surgeries, n (%)	444 (83.8%)	117 (79.1%)	327 (85.6%)	0.07	20 (57.1%)	424 (85.7%)	<0.0001
Anatomic differences, n (%)	389 (73.5%)	95 (64.6%)	294 (77.0%)	0.004	17 (48.6%)	372 (75.3%)	0.001
Diabetes, n (%)	92 (17.4%)	32 (21.6%)	60 (15.7%)	0.11	6 (17.1%)	86 (17.4%)	0.97
Moderate to severe kidney disease, n (%)	25 (4.7%)	5 (3.4%)	20 (5.2%)	0.37	4 (11.4%)	21 (4.2%)	0.07*
Leukemia or lymphoma, n (%)	16 (3.0%)	3 (2.0%)	13 (3.4%)	0.57*	3 (8.6%)	13 (2.6%)	0.08*
Peptic ulcer disease, n (%)	18 (3.4%)	5 (3.4%)	13 (3.4%)	0.99	0 (0.0%)	18 (3.6%)	0.62*
*Visit measures*							
Triage heart rate >110 bpm, n (%)	42 (8.0%)	16 (10.9%)	26 (6.8%)	0.12	4 (11.4%)	38 (7.7%)	0.51*
Triage respiratory rate >20, n (%)	23 (4.4%)	5 (3.4%)	18 (4.7%)	0.50	6 (17.1%)	17 (3.5%)	0.003*
Triage SBP <100 mm Hg, n (%)	28 (5.3%)	12 (8.1%)	16 (4.2%)	0.07	3 (8.6%)	25 (5.1%)	0.42*
Triage temp >100.4 °F, n (%)	4 (0.8%)	3 (2.1%)	1 (0.3%)	0.07*	1 (3.0%)	3 (0.6%)	0.23*
Triage O_2_ <90%, n (%)	10 (1.9%)	4 (2.7%)	6 (1.6%)	0.48*	2 (5.7%)	8 (1.6%)	0.14*
Nasogastric decompression, n (%)	342 (64.5%)	106 (71.6%)	236 (61.8%)	0.03	24 (68.6%)	318 (64.2%)	0.60
Gastrografin administration, n (%)	83 (15.7%)	14 (9.5%)	69 (18.1%)	0.01	7 (20.0%)	76 (15.4%)	0.46
Focal tenderness on examination, n (%)	206 (38.9%)	57 (38.5%)	149 (39.0%)	0.55	13 (37.1%)	193 (39.0%)	0.97
Rebound tenderness/peritonitis on examination, n (%)	13 (2.5%)	3 (2.0%)	10 (2.6%)	0.90	0 (0.0%)	13 (2.6%)	0.14
Distension on examination, n (%)	228 (43.0%)	62 (41.9%)	166 (43.5%)	0.39	19 (54.3%)	209 (42.2%)	0.24
Diffuse tenderness on examination, n (%)	277 (52.3%)	73 (49.3%)	204 (53.4%)	0.14	16 (45.7%)	261 (52.7%)	0.64
*CT findings*							
Presence of a transition point on CT, n (%)	475 (95.0%)	134 (97.1%)	341 (94.2%)	0.18	30 (88.2%)	445 (95.5%)	0.08
Presence of free intraperitoneal fluid on CT, n (%)	278 (55.5%)	80 (57.6%)	198 (54.7%)	0.56	22 (64.7%)	256 (54.8%)	0.26
Debris and gas bubbles within the dilated small bowel lumen on CT, n (%)	174 (35.2%)	37 (27.4%)	137 (38.2%)	0.03	8 (23.5%)	166 (36.1%)	0.13
Mesenteric edema on CT, n (%)	183 (36.8%)	53 (38.4%)	130 (36.1%)	0.63	11 (32.4%)	172 (37.1%)	0.58
Volvulus or closed-loop obstruction on CT, n (%)	61 (12.2%)	42 (30.4%)	19 (5.2%)	<0.0001	5 (14.7%)	56 (12.0%)	0.59*

* P-value calculated by the Fisher exact test.

*CCI*, Charlson Coborbidity Index; *SBO*, small bowel obstruction; *CT*, computed tomography; *bpm*, beats per minute; *SBP*, systolic blood pressure; *mm Hg*, millimeters of mercury; *O*_*2*_, oxygen saturation.

**Table 2.** Occurrence of outcomes.

Outcome	N (%)
Surgery	148 (27.9%)
Complication	35 (6.6%)
Sepsis	16 (3.0%)
Intubation	12 (2.2%)
Vasopressor dependent shock	11 (2.0%)
Anatomic surgical alteration	1 (0.2%)
Bowel perforation	8 (1.5%)

We identified univariate associations of the decision for surgical intervention (Table 1). In our multivariable analysis, we identified three independent factors associated with a higher odd of surgical intervention: fluid or volvulus/closed-loop obstruction on CT (OR 7.95, 95% CI 4.25–15.39), nasogastric decompression (OR 2.04, 95% CI 1.25–3.39), and initial systolic blood pressure <100 millimeters of mercury (OR 2.65, 95% CI 1.05–6.53) (Table 3). Four factors were associated with lower odds of surgical intervention: Higher CCI score (OR 0.87, 95% CI 0.80–0.95), abdominal distension in history (OR 0.27, 95% CI 0.10–0.62), previous SBO (OR 0.42, 95% CI 0.26–0.66), and gastrografin administration (OR 0.41, 95% CI 0.20–0.81). The AUC for the logistic regression was 0.73.

**Table 3.** Results of multivariable logistic regression analysis for decision for surgery.

Variable	Odds ratio (95% CI)
History of abdominal distension	0.27 (0.10–0.62)
Gastrografin	0.41 (0.20–0.81)
Previous SBO	0.42 (0.26–0.66)
Higher CCI score	0.87 (0.80–0.95)
Nasogastric decompression	2.04 (1.25–3.39)
Initial systolic blood pressure <100 mm Hg	2.65 (1.05–6.53)
Free fluid or volvulus/closed-loop obstruction on CT	7.95 (4.25–15.39)

*CI*, confidence interval; *SBO*, small bowel obstruction; *CCI*, Charlson Comorbidity Index; *mm HG*, millimeters of mercury; *CT*, computed tomography.

## DISCUSSION

The general treatment method of SBOs is shifting toward non-operative management. While there have been attempts to construct better guidelines such as the Bologna guidelines, there is still no definitive consensus.^5^ We propose that these clinical decision algorithms can be strengthened by incorporating specific factors. An example of a simple predictor model is seen in the retrospective study by Komatsu et al, where they created a four-point system scoring the risk for surgery in patients undergoing conservative strategy with their three variables.^11^ Past studies regarding surgical associations for SBOs have mixed results. Free fluid on CT was found as a positive predictor for surgery in some studies.^3^ ^,^ ^6^ ^,^ ^12^ However, a later prospective validation study found that free fluid was not a predictor.^13^

Our results demonstrated that free fluid or volvulus/closed-loop obstruction was positively associated with an OR of 7.95 (95% CI 4.25–15.39). It is important to note that our result is a combination of two distinct variables; we found that one was found to be completely predictive of the other and, therefore, we merged the two variables. Another CT finding, namely mesenteric edema has previously been attributed as a positive predictor.^13^ Our results, however, did not identify mesenteric edema as significant in the univariant analysis and was not included in the multivariant model.

Interestingly, we found prior SBO to be an independent association, which is protective for the decision for surgery with an OR of 0.42 (95% CI 0.26–0.66). In both Zielinski et al studies prior SBOs were seen as protective for operative management in their univariate analysis, but it was not significant in their regression models.^3^ ^,^ ^13^ Prior SBO was also found to be significant in the bivariate analyses in the O’Daly et al study but was not significant in their regression model.^12^ This is in contrast to the results of another study that did not find prior SBOs to be significant in their univariate analysis with a *P*-value of 0.93.^6^ A possible explanation for why prior SBOs is protective in the decision for surgery is that there might be a higher risk of recurrence in patients who received conservative treatment, while the need for surgical re-intervention was not different between non-surgical and surgical management.^7^ This coupled with a possible bias on the clinician’s part that if a previous management worked the clinician might favor it again, would influence the number of patients who received conservative treatment in our study time frame. There will simply be more patients with previous SBOs that have been treated conservatively in that given time.

Gastrografin has recently been included in some evidence-based papers regarding SBO management and decision-making.^5^ ^,^ ^14^ It is a hyperosmolar water-soluble contrast medium shown to have a diagnostic and prognostic, as well as a potential therapeutic, effect for patients with SBO.^15^ ^–^ ^17^ The prognostic and therapeutic properties of gastrografin have been appreciated during a gastrografin challenge, when the patient ingests gastrografin either orally or through a nasogastric tube, and multiple radiographs are taken in the span of 24 hours to assess passage of contrast. Three large systematic reviews and meta-analyses have determined when gastrografin has passed through the patient’s gastrointestinal tract and reached the colon within 24 hours, the patient most likely does not need surgery and can, therefore, be treated conservatively. Additionally, all three studies found that the admission of gastrografin shortened the duration of hospitalization.^15^ ^–^ ^17^

We found that gastrografin was an independent negative association for surgery (OR 0.41, 95% CI 0.20–0.81). While there may be selection bias for less acutely ill patients, it is hypothesized that there is a therapeutic effect of gastrografin, which may decrease the need for surgery. A large systematic review and meta-analysis involving 1,216 patients from 12 studies found the OR for surgery intervention after gastrografin administration to be 0.55 (95% CI 0.32–0.37, *P* = 0.003)^15^. This is in line with another systematic review and meta-analysis finding the OR to be 0.62 (95% CI 0.44–0.88, *P* = 0.007)^16^.

## LIMITATIONS

The primary limitation to our study is its retrospective, single-center design, which left us open to selection bias. Another limitation is that surgeon practice variability may not be generalizable to other institutions. In addition, our chart review methodology to collecting data inherently made us susceptible to misclassification bias. It is also possible that patients may have re-presented to another hospital without our knowledge and those potential complications and outcomes were lost to follow-up. Our study is further limited by our small sample size, and we were unable to incorporate all variables and complications we found significant into the multivariant model. Additionally, by using surgery as an endpoint, we may have captured patients who received surgery but did not unequivocally need surgery. Finally, we did not classify which patients were admitted to a surgical service as that may have impacted the decision to perform surgery if they were not on a surgical service.

## CONCLUSION

We found seven independent factors associated with surgery. We believe that these may help clinicians determine which ED patients require surgical intervention. There are conflicting results in the current literature and further research is needed to determine more accurate algorithms and patient management of patients with small bowel obstruction. We believe our larger retrospective study provides an important advancement in potentially formulating better prediction models to prevent unnecessary surgery. This research is imperative for the management of SBOs, as unnecessary surgery yields a higher resource utilization and can lead to further complications.^2^ ^–^ ^4^ ^,^ ^7^

Future studies should include large, prospective, multi-institutional studies encompassing a wide range of variables and CT parameters, as current literature is limited. Additional prediction models and algorithms should be tested in combination with the administration of gastrografin to build a comprehensive management plan for patients with SBO.
